# Publisher's Note

**DOI:** 10.1093/pcmedi/pbac012

**Published:** 2022-05-13

In April 2022, three articles intended for the journal *Psychoradiology* were mistakenly published into *Precision Clinical Medicine* Volume 1, Issue 1, June 2018.

The articles were:

https://doi.org/10.1093/psyrad/kkaa001

https://doi.org/10.1093/psyrad/kkab001

https://doi.org/10.1093/psyrad/kkaa002

These articles have now been moved to *Psychoradiology*, Volume 1, Issue 1, March 2021. The publisher apologizes for this error.

